# Polyomaviruses shedding in stool of patients with hematological disorders: detection analysis and study of the non-coding control region’s genetic variability

**DOI:** 10.1007/s00430-019-00630-9

**Published:** 2019-08-02

**Authors:** Carla Prezioso, Marco Ciotti, Francisco Obregon, Donatella Ambroselli, Donatella Maria Rodio, Laura Cudillo, Javid Gaziev, Annamaria Mele, Angelo Nardi, Cartesio Favalli, William Arcese, Anna Teresa Palamara, Valeria Pietropaolo

**Affiliations:** 1 Department of Public Health and Infectious Diseases, “Sapienza” University, P.le Aldo Moro, 5, 00185 Rome, Italy; 2 Laboratory of Clinical Microbiology and Virology, Polyclinic Tor Vergata Foundation, Rome, Italy; 3 Stem Cell Transplant Unit, Polyclinic Tor Vergata Foundation, Rome, Italy; 4 International Center for Transplantation in Thalassemia and Sickle Cell Anemia, Mediterranean Institute of Hematology, Polyclinic Tor Vergata Foundation, Rome, Italy; 5 Catholic University “Our Lady of Good Counsel”, Laprake, Rruga Dritan Hoxha, Tirana, Albania; 6 Department of Public Health and Infectious Diseases, Institute Pasteur, Cenci-Bolognetti Foundation, Sapienza University of Rome, Rome, Italy; 7 San Raffaele Pisana Scientific Institute for Research, Hospitalization and Health Care, Rome, Italy

**Keywords:** HPyVs, Hematological patients, Stool samples, Gastrointestinal tract, NCCR/VP1, Phylogenetic analysis

## Abstract

Fragmented data are available on the human polyomaviruses (HPyVs) prevalence in the gastrointestinal tract. Rearrangements in the non-coding control region (NCCR) of JCPyV and BKPyV have been extensively studied and correlated to clinical outcome; instead, little information is available for KIPyV, WUPyV and MCPyV NCCRs. To get insights into the role of HPyVs in the gastrointestinal tract, we investigated JCPyV, BKPyV, KIPyV, WUPyV and MCPyV distribution among hematological patients in concomitance with gastrointestinal symptoms. In addition, NCCRs and VP1 sequences were examined to characterize the strains circulating among the enrolled patients. DNA was extracted from 62 stool samples and qPCR was carried out to detect and quantify JCPyV, BKPyV, KIPyV, WUPyV and MCPyV genomes. Positive samples were subsequently amplified and sequenced for NCCR and VP1 regions. A phylogenetic tree was constructed aligning the obtained VP1 sequences to a set of reference sequences. qPCR revealed low viral loads for all HPyVs searched. Mono and co-infections were detected. A significant correlation was found between gastrointestinal complications and KIPyV infection. Archetype-like NCCRs were found for JCPyV and BKPyV, and a high degree of NCCRs stability was observed for KIPyV, WUPyV and MCPyV. Analysis of the VP1 sequences revealed a 99% identity with the VP1 reference sequences. The study adds important information on HPyVs prevalence and persistence in the gastrointestinal tract. Gastrointestinal signs were correlated with the presence of KIPyV, although definitive conclusions cannot be drawn. HPyVs NCCRs showed a high degree of sequence stability, suggesting that sequence rearrangements are rare in this anatomical site.

## Introduction

Human polyomaviruses (HPyVs) are small non-enveloped DNA viruses with a circular double-stranded DNA genome divided into three functional regions: early, late and, interposed between these regions, the non-coding control region (NCCR) [1]. HPyVs have a worldwide distribution and each member has a restrict host range. JC virus (JCPyV), isolated in 1971 from the brain of a patient with Hodgkin’s disease, was the first HPyV described [2]. In the same year, BK virus (BKPyV) was isolated from the urine sample of a renal transplant patient [3]. Since then, other HPyVs have been identified including KI polyomavirus (KIPyV) [4] and WU polyomavirus (WUPyV) [5], isolated from respiratory samples, and Merkel cell polyomavirus (MCPyV) identified in Merkel cell carcinoma (MCC) [6]. Following primary infection, which occurs usually in childhood through the respiratory or oral route, HPyVs persist in the body in a latent state [7]. Reactivation is frequent in immunocompromised patients with possible severe consequences [7, 8]. JCPyV can cause progressive multifocal leukoencephalopathy (PML) [9], whereas BKPyV is associated with hemorrhagic cystitis and interstitial nephropathy in transplant patients [10]. MCPyV is associated with MCC, a rare and aggressive skin cancer [6]. Conversely, disease association has not been documented for KIPyV and WUPyV [11]. Both viruses were detected in the respiratory secretions of children with acute respiratory symptoms [4, 5, 11] and rarely in those with gastrointestinal disorders [4, 12].

Prevalence studies detected HPyVs in stool specimens of hospitalized children [13–16] and JCPyV and BKPyV also in feces of healthy adults [17, 18]. Furthermore, reactivation and gastrointestinal excretion of KIPyV and WUPyV have been reported in immunosuppressed transplant patients [19]. Environmental virology studies detected HPyVs in sewage samples and polluted water supporting the possibility of an oral–fecal route of spread [7, 20–22]. Despite these studies, the data on the prevalence of HPyVs in the gastrointestinal tract are still scarce.

Concerning the NCCR’s genetic variability, while rearrangements in the NCCR of JCPyV and BKPyV have been extensively studied and linked to clinical outcomes [23–25], little information is available on KIPyV, WUPyV and MCPyV NCCRs [26, 27].

To get insight into the role played by HPyVs in the gastrointestinal tract, we investigated the distribution of JCPyV, BKPyV, KIPyV, WUPyV and MCPyV in a group of patients with hematological disorders. The presence of the viruses was evaluated in concomitance with the gastrointestinal symptoms, and the genetic characterization of the strains circulating among patients was performed analyzing the VP1 and NCCR sequences.

## Materials and methods

### Patients, sample collection and processing

The study includes 62 transplanted patients with hematological disorders afferent to the University Hospital Tor Vergata (Rome, Italy). Patients were divided into two sub-groups with respect to the disease: a sub-group of 31 thalassemic patients (15 females and 16 males; mean age: 9.25 years; median age: 9 years) and a sub-group of 31 patients affected by acute myeloid leukemia (AML) (16 females and 15 males, mean age: 41.38 years; median age: 38 years). A total of 62 stool samples was collected from April to December 2018. Signed informed consent was obtained in accordance with the Declaratio7n of Helsinki and the Ethic Committee guidelines (RTN-CL-MOD020).

Total DNA was extracted from 0.2 mg of stool using the Stool DNA Isolation Kit following the manufacturer’s instructions (NORGEN BIOTEK, Canada). The extraction and quality of nucleic acid were checked amplifying the human β-globin gene as previously described [28].

### Screening of HPyVs DNA by quantitative real-time PCR

Quantitative amplification assay (qPCR) was carried out to detect and quantify JCPyV, BKPyV, KIPyV, WUPyV, MCPyV DNA, using the 7300 Real-Time PCR System (Applied Biosystems, CA, USA) and published protocols [6, 29–31]. All samples were tested in triplicate and the number of viral copies was calculated from an external standard curve obtained using serial dilutions at known titer (range: 10^2^–10^5^ copies) of JCPyV pCY/cl1 plasmid (ATCC^®^VRMC-1™), WUPyV pcDWUER (#37093) and KIPyV pcDKIER (#37094) plasmids, and MCPyV pcDNA6 sTco plasmid (#40201) (Addgene, Cambridge, MA). Quantitative determination of BKPyV DNA was performed using Thermo Scientific™ AcroMetrix™ BKPyV Panel containing intact, encapsidated viral particles (VP1). Standard precautions were taken to prevent contamination during amplification procedures. The lower detection limit of the assay was 10 DNA copies of the target gene per amplification reaction, corresponding to 10 genome equivalents per reaction (10 gEq/reaction).

### Amplification, sequencing and analysis of HPyVs NCCRs

All HPyVs DNA-positive samples were subsequently amplified for NCCR following published protocols [21, 32–34], on the 9700 GeneAmp^®^ PCR System (AB Applied Biosystems, CA, USA). The amplified products were purified using the MinElute PCR Purification Kit (QIAGEN, Milan, Italy) and sequenced in a dedicated facility (Bio-Fab research s.r.l., Rome, Italy). The obtained sequences were compared to reference sequences deposited in GenBank (AB081613, AB263926, EF127906, EF444549, EU375803). Sequence alignments were performed with ClustalW2 at the European Molecular Biology Laboratory–European Bioinformatics Institute (EMBL-EBI) website using default parameters [35].

### Amplification, sequencing and analysis of HPyVs VP1 regions

All HPyVs DNA-positive samples were also subjected to PCR amplification of the VP1 region, following published protocols [4, 5, 34, 36, 37]. The amplicon size was as follows: BKPyV, 327 bp; JCPyV, 215 bp; KIPyV, 207 bp; WUPyV, 369 bp; MCPyV, 589 bp.

The amplified products were purified using the MinElute PCR Purification Kit (QIAGEN, Milan, Italy) and sequenced in a dedicated facility (Bio-Fab research s.r.l., Rome, Italy). BKPyV subtypes/sub-groups and JCPyV genotypes/subtypes were classified based on the single nucleotide polymorphisms found within the amplified VP1 region. Sequence alignment for all HPyVs VP1 isolates were performed with ClustalW2 at the EMBL-EBI website using default parameters [35].

### Amplification and sequencing of reference strains

The quality of our sequences was also checked amplifying the VP1 and NCCR regions of the reference strains AB081613, AB263926, EF127906, EF444549, EU375803 cloned into a luciferase reporter plasmid. PCR conditions were the same as for the DNA extracted from stool samples. Amplicons processing and sequences analyses were as reported above.

### HPyVs phylogenetic analysis

A phylogenetic tree was generated using Molecular Evolutionary Genetics Analysis (Mega) version 6.0 software program [38] after aligning the VP1 sequences isolated from the patients to those of the reference strains: JCPyV AB081613, BKPyV AB263926, KIPyV EF127906, WUPyV EF444549, MCPyV EU375803. A bootstrap test with 1000 replicates was performed to evaluate the confidence of the branching pattern of the tree.

### Statistical analysis

HPyVs detection was summarized by counts and proportions. If continuous variables were normally distributed, they were expressed as mean ± SD, if not, they were expressed by median and range. The χ2 test was performed to evaluate differences in the viral detection among patient groups, while Mann–Whitney *U* test was applied for non-normally distributed continuous variables to analyze differences between patients. A *p* value below 0.05 was considered statistically significant.

## Results

### HPyVs detection

KIPyV, WUPyV, BKPyV, JCPyV and MCPyV DNAs were detected in both thalassemic and leukemic patients, although with different prevalences. Overall, KIPyV DNA was found in 20/62 samples (32.3%), WUPyV DNA in 20/62 (32.3%), BKPyV DNA in 20/62 (32.3%), JCPyV DNA in 29/62 (47%) and MCPyV DNA in 25/62 (40%). qPCR results showed low amounts of viral DNA with an average value of 5 × 10^2^ gEq/mg (95% CI 4.3–5.8) for KIPyV, 1.5 × 10^2^ gEq/mg (95% CI 1–2) for WUPyV, 6.3 × 10^2^ gEq/mg (95% CI 6.2–6.5) for BKPyV, 7 × 10^2^ gEq/mg (95% CI 6.9–7.2) for JCPyV, and 4.5 × 10^2^ gEq/mg (95% CI 3–8) for MCPyV.

### HPyVs mono- and co-infections

**Fig. 1:**
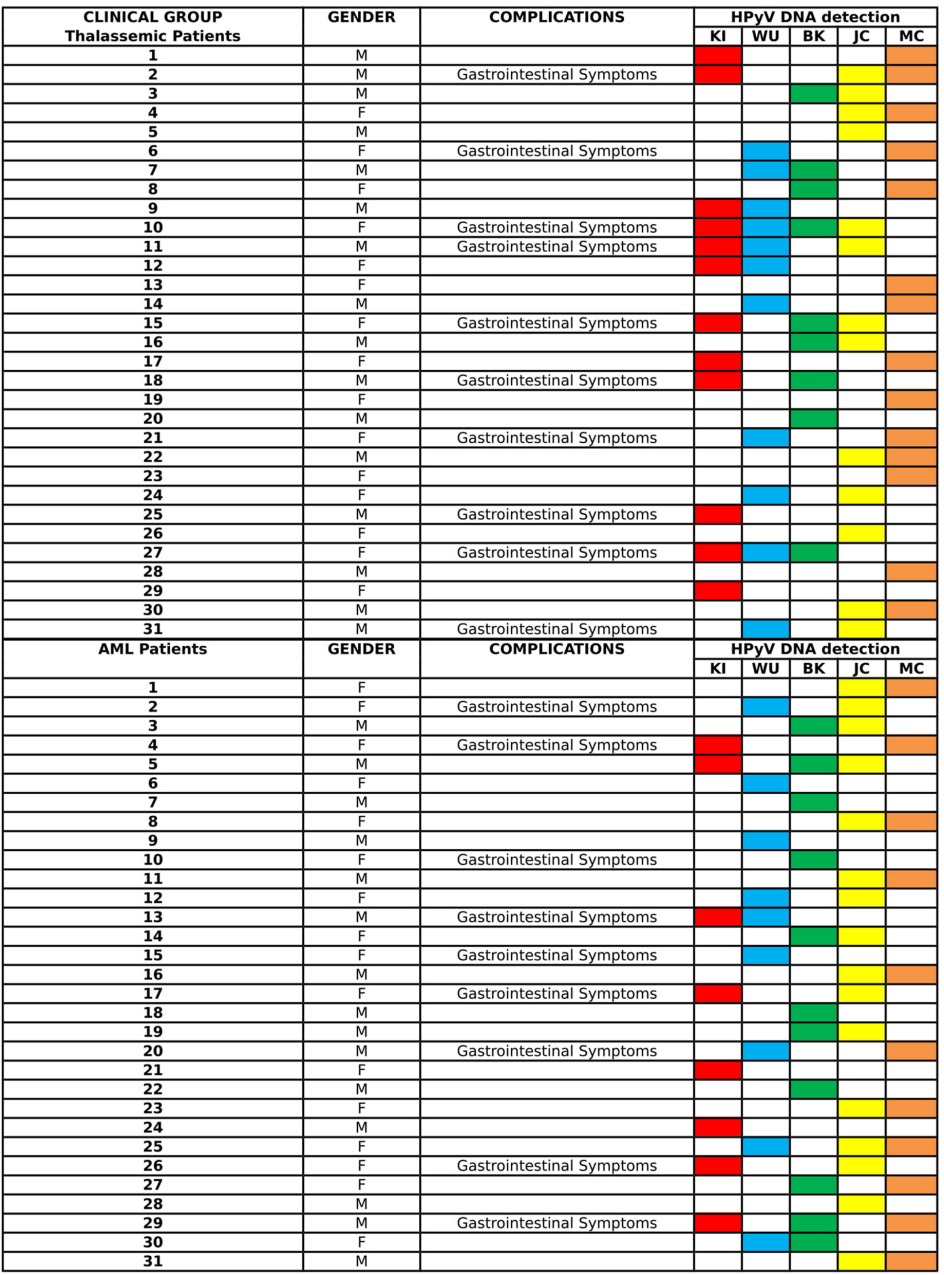
The figure summarizes the detection, as single or multiple infections, of the five HPyVs investigated in this study. Patients who developed gastrointestinal symptoms are also displayed

Considering HPyVs mono-infection, KIPyV DNA was detected in 12/31 thalassemic patients (6 females/6 males) and in 8/31 (4 females/4 males) AML patients. WUPyV DNA was identified in 11/31 thalassemic patients (6 females/5 males) and in 9/31 AML patients (6 females/3 males). BKPyV DNA was detected in 9/31 thalassemic patients (4 females/5 males) and 11/31 AML patients (4 females/7 males). JCPyV DNA was present in 13/31 thalassemic (8 females/5 males) and in 16/31 AML patients (9 females/7 males). Finally, MCPyV DNA was found in 14/31 thalassemic patients (8 females/6 males) and in 11/31 AML patients (6 females/5 males) (Fig. 1). Examining HPyVs co-infection patterns, the most frequent combination was JCPyV/MCPyV, found in nine samples (three from thalassemic patients and six from patients with AML). All co-infections are summarized in Fig. 1.

No significant association was found between the presence of HPyV DNA and age or gender (Table 1). Instead, a significant association was found between the presence of gastrointestinal symptoms and KIPyV infection, both in thalassemic (*p* = 0.014) and AML patients (*p* = 0.015) (Table 1).

**Table 1:** Analysis of HPyVs detection in relation to age, gender and gastrointestinal symptoms

Thalassemic patients	HPyVs-positive samples	Age	*p* value
31 [15 F, 16 M]	31	2–18 (mean age: 9.25 years; median age: 9 years)	*p* ≥ 0.05

Thalassemic patients	HPyVs-positive samples	Gender	*p* value
31	31	[15 F, 16 M]	*p* ≥ 0.05

Thalassemic patients	KIPyV-positive samples	Gastrointestinal symptoms	*p* value
31	12	7/12	*p* = 0.014

AML patients	HPyVs-positive samples	Age	*p* value
31 [16 F; 15 M]	31	25–65 (mean age: 41.38 years; median age: 38 years)	*p* ≥ 0.05

AML patients	HPyVs-positive samples	Gender	*p* value
31	31	[16 F, 15 M]	*p* ≥ 0.05

AML patients	KIPyV-positive samples	Gastrointestinal symptoms	*p* value
31	8	5/9	*p* = 0.015

*HPyVs* human polyomaviruses, *AML* acute myeloid leukemia

### Sequencing analysis of HPyVs NCCRs

**Fig. 2:**
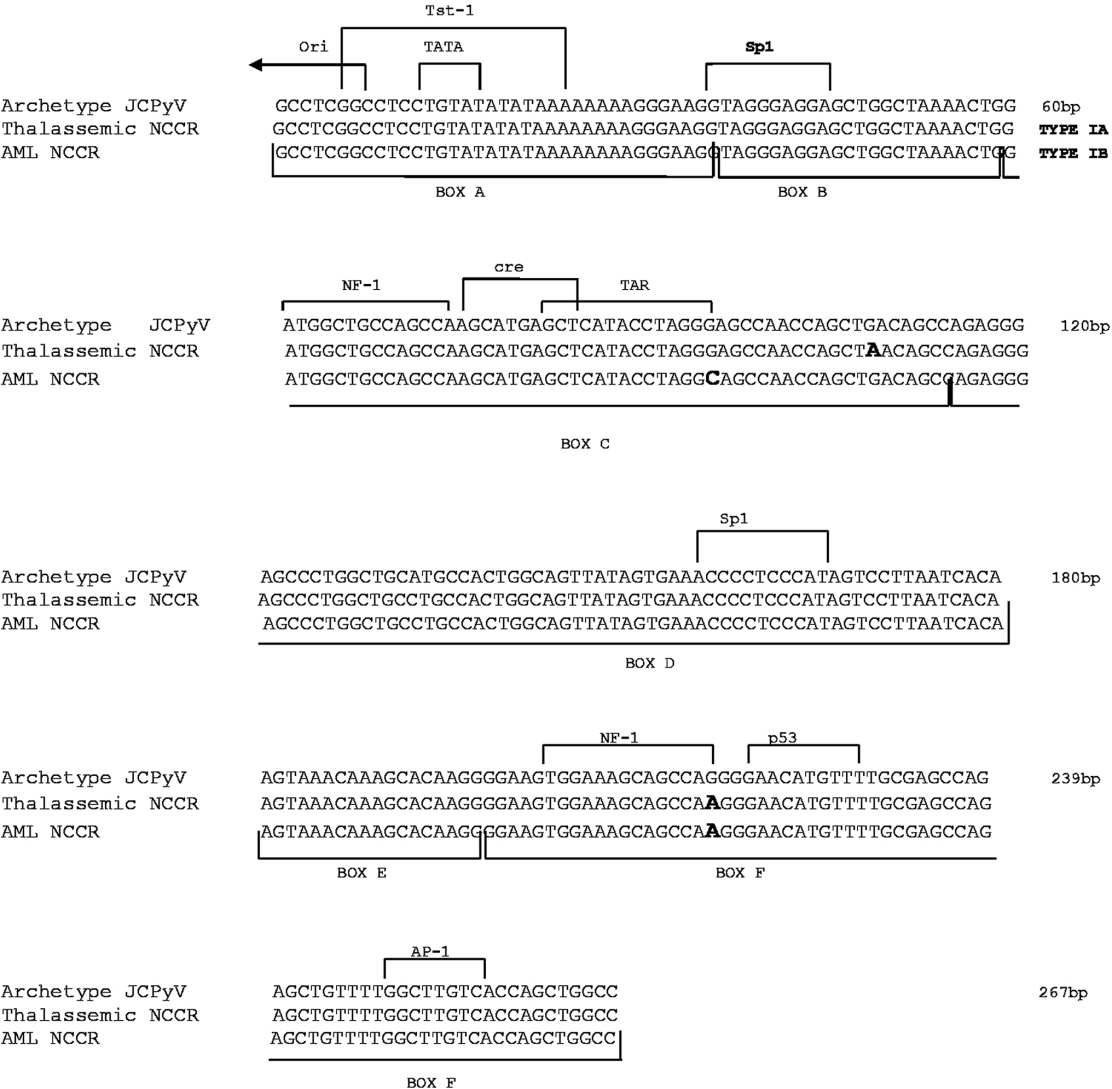
Analysis of the NCCR structure organization of JCPyV. Alignment of JCPyV non-coding control region (NCCR) isolates from stool specimens of thalassemic and AML patients revealed an archetype CY-like NCCR, with the conservative boxes A, B, C, D, E and F. The NCCR sequence of the archetypal JCPyV strain CY, as proposed by Yogo et al. [39] is shown at the top of the figure and the consensus sequences of the NCCR showing variations isolated from thalassemic and AML patients are shown below. The identified genotype is reported at the top of the figure, right side. The nucleotide changes G95C, G108A and G217A identified in the patients’ isolates are in bold. The six boxes commonly used to divide the archetypal NCCR [39] are indicated under the NCCR sequences, while proven and putative binding sites for transcriptional factors are reported above the CY NCCR sequence

Sequence analysis of 29 JCPyV NCCRs revealed an archetype CY-like NCCR, with the conservative regions A, B, C, D, E and F [39] in all samples. However, single nucleotide variations were observed in two specimens: G95C and G217A in an AML patient, and G108A and G217A in a thalassemic patient. The G95C and G217A involve the cellular transcription-binding sites TAR and NF1, respectively, while the G108A falls outside transcription-binding sites (Fig. 2).

**Fig. 3:**
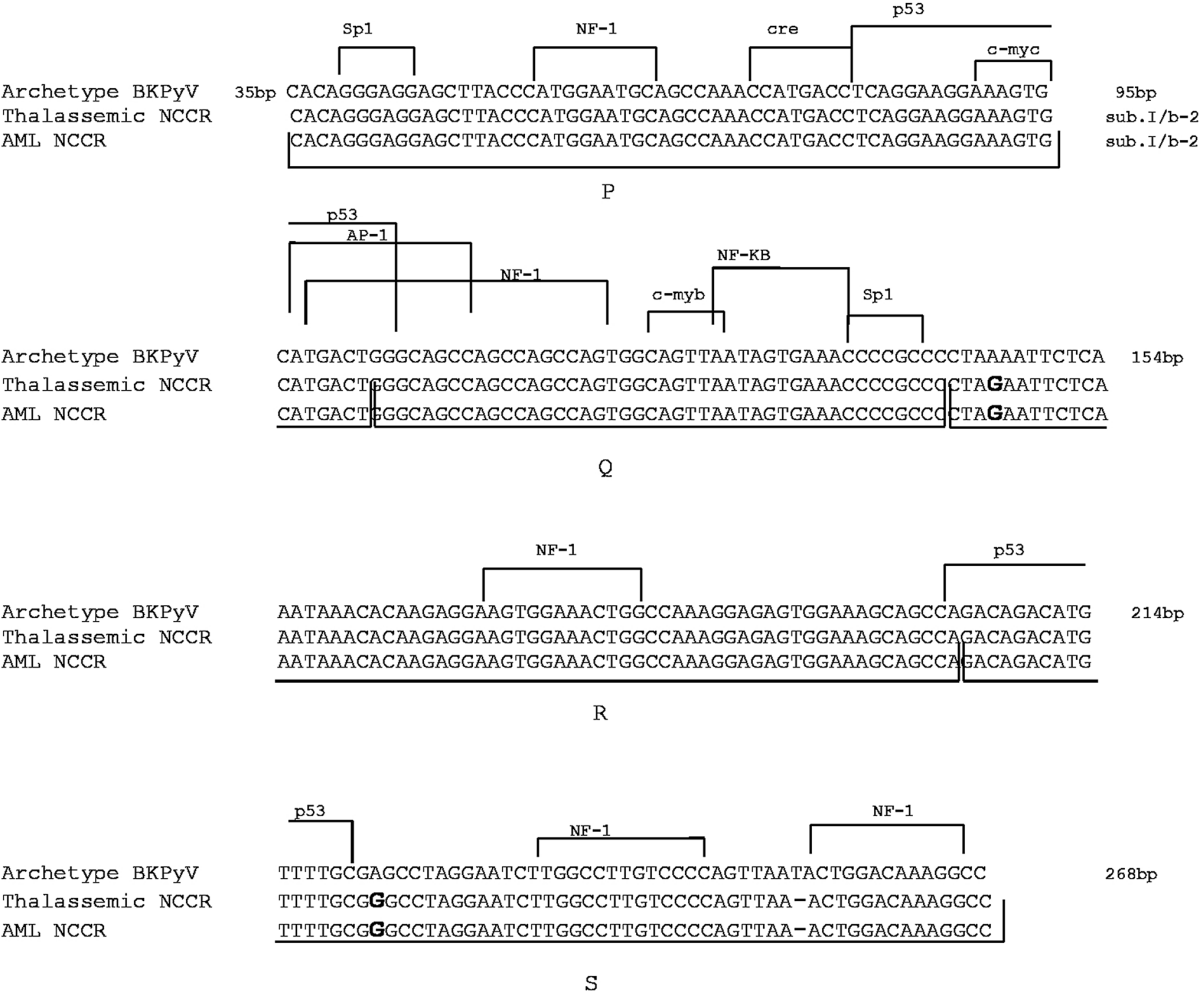
Analysis of the NCCR structure organization of BKPyV. Alignment of BKPyV non-coding control region (NCCR) isolates from stool specimens of thalassemic and AML patients revealed an archetype NCCR sequence. Blocks P, Q, R and S commonly used to denote the archetypal NCCRs [40, 41] are indicated under the NCCR sequences, while proven and putative binding sites for transcriptional factors are reported above the archetypal NCCR sequence. The O-block, containing the origin of replication, was omitted. The archetype NCCR is shown at the top of the figure. The nucleotide sequence variations identified at positions 4 (R-block) and 18 (S-block) are in bold. The thymidine deletion at nucleotide position 50 (S-block) is indicated with a dash line. The genotype/subtype identified in the patients’ isolates is reported at the top of the figure, right side

NCCRs amplified from 20 BKPyV-positive samples were compared to the archetype BKPyV NCCR sequence, arbitrarily divided into O, P, Q, R, and S blocks [40, 41]. All BKPyV analyzed regions were identical to the archetype NCCR. In one thalassemic patient and in one AML patient, point mutations at nucleotide positions 4 (R-block) and 18 (S-block) and a thymidine deletion at nucleotide position 50 (S-block) were identified. All mutations were located outside the cellular transcriptional factor-binding sites (Fig. 3). Regarding KIPyV NCCRs sequences, no nucleotide variations were detected in our KIPyV-positive samples. Sequences were identical to Stockholm 60 reference sequence (GenBank: EF127906) [4]. Finally, NCCRs recovered from 20 WUPyV- and 25 MCPyV-positive samples were identical to those deposited in GenBank under the accession numbers EF444549 and EU375803 [5, 6], except for some nucleotide variations already described [34, 42] (data not shown). Overall, sequence similarity with WUPyV and MCPyV reference strains ranged from 99.5 to 100%.

To confirm that HPyVs NCCR mutations did not originate from DNA polymerase-induced mistakes, we also amplified the NCCRs of the reference strains AB081613, AB263926, EF127906, EF444549 and EU375803, and sequenced them. Analysis of the sequences revealed that they were identical to those of the reference strains, indicating that the PCR did not introduce mutations.

### Analysis of HPyVs VP1 region

Sequences analysis of KIPyV, WUPyV and MCPyV VP1 amplicons showed 1–3 nucleotides difference with respect to the reference strains EF127906, EF444549, EU375803, although these variations did not produce any amino acid change in the derived protein sequence. Among the 29 JCPyV-positive samples, there was a prevalence of the European genotypes 1A and 1B. Instead, all BKPyV-positive samples contained the BKPyV subtype I/subgroup b-2.

### HPyVs phylogenetic analysis

**Fig. 4:**
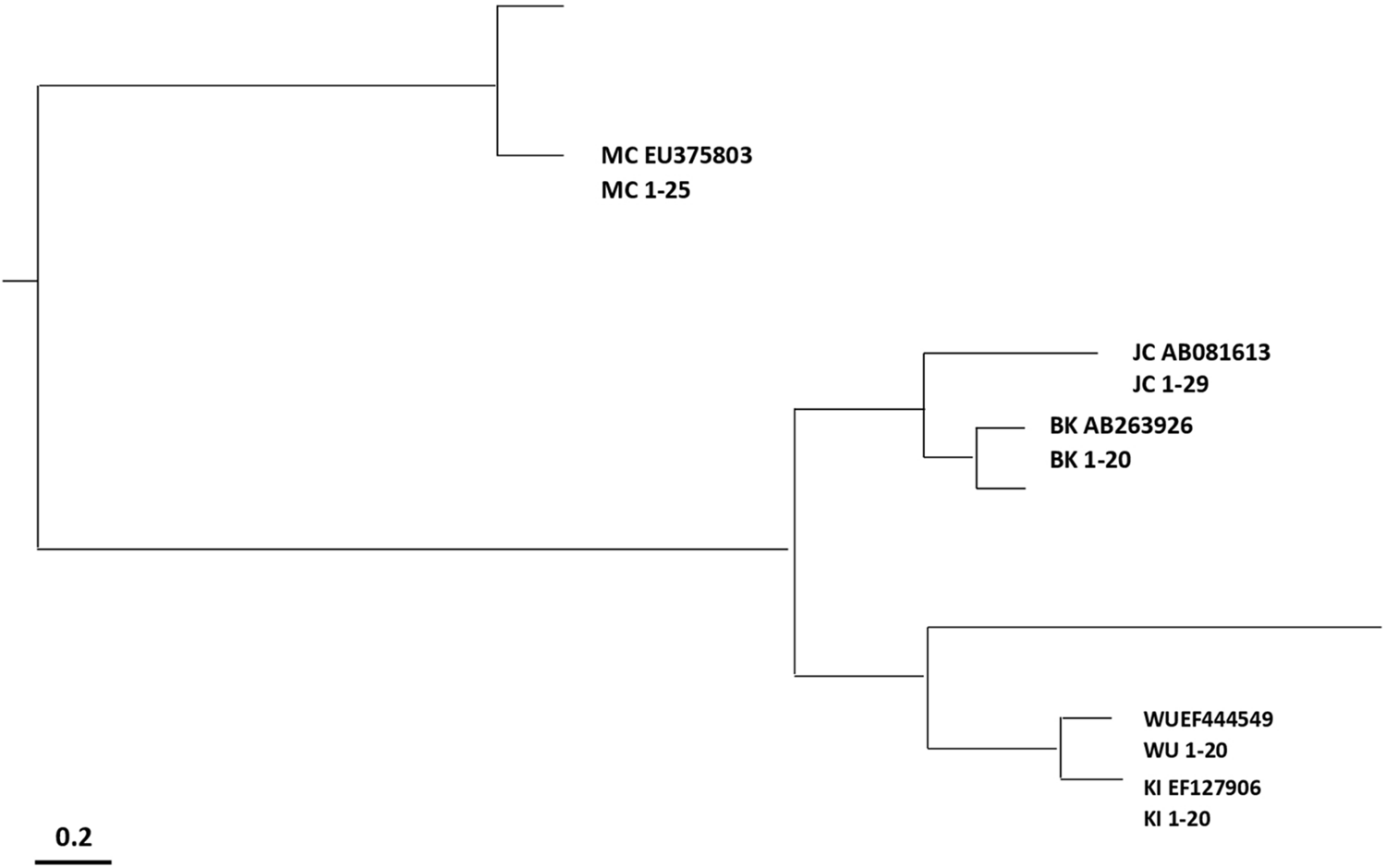
HPyVs phylogenetic analysis. Phylogenetic tree generated using MEGA version 6 software. The VP1 gene sequences obtained from the patients’ isolates cluster with the corresponding reference sequences: JCPyV AB081613, BKPyV AB263926, KIPyV EF127906, WUPyV EF444549 and MCPyV EU375803. The bar at the bottom of the figure indicates 0.2 nucleotide substitutions per site

The phylogenetic analysis carried out on the VP1 sequences obtained from the thalassemic and AML patients revealed that the isolates were 99% identical to the reference sequences (JCPyV AB081613, BKPyV AB263926, KIPyV EF127906, WUPyV EF444549, MCPyV EU375803). All isolates of each investigated virus clustered together and with the corresponding reference strains (Fig. 4).

## Discussion

Infections in transplanted patients are related to the kinetics of immune reconstitution and are frequently due to reactivation of latent viruses [19]. BKPyV and JCPyV reactivation in hematological transplanted patients are a common observation because of the immunosuppressive status induced by the conditioning regimen and/or immunosuppressive drugs administered after transplant. For this reason, in the post-transplant phase, BKPyV and JCPyV are regularly monitored by qPCR in urine and blood. The presence of both viruses in these compartments, along with a progressive increase in viral load, may be associated with hemorrhagic cystitis [43], or an increased risk of developing PML [7], respectively. While the association with these diseases has been established, few and fragmented data are currently available about the persistence and excretion of BKPyV and JCPyV as well as KIPyV, WUPyV and MCPyv in the gastrointestinal tract. Our data provides evidence that HPyVs are shed in stool specimens of hematological patients, although at low level, supporting the hypothesis that gastrointestinal tract may be a site of HPyVs persistence and reactivation in humans.

There was no association between polyomaviruses shedding in fecal matter and gender as well as HPyVs shedding in fecal matter and age as reported also by Vanchieri et al. [44].

Among the HPyVs investigated, JCPyV and MCPyV were the polyomaviruses most frequently detected. The higher detection rate of the two polyomaviruses compared to the others could be explained by the wide circulation of JCPyV and MCPyV among the populations [45] or by a higher excretion rate of the two HPyVs.

Several studies have examined the prevalence of HPyVs in stool samples with conflicting results. Bergallo et al. [46] reported detection rates for KIPyV (31%) and WUPyV (25%) similar to ours, while a lower prevalence was reported by other authors [47–50]. Detection rate ranged from 2.7 to 11% for KIPyV [48, 49] and from 0.9 to 14% for WUPyV [47–50]. Similarly, the prevalence reported in this study for MCPyV, BKPyV and JCPyV differs from that reported in other studies. In our leukemic patients, MCPyV was detected at a higher rate compared to the study of Kantola et al. [49] who investigated the prevalence of MCPyV in stool samples from leukemic children. The difference in the age group might explain these results. Also, the detection rate of BKPyV and JCPyV is much higher than that reported among healthy adults [44], but similar to that found in hematopoietic stem cell transplant patients, strengthening the hypothesis that immunosuppression increases fecal shedding and the gastrointestinal tract may be a site of latency of HPyVs [51].

After transplantation, gastrointestinal symptoms may be caused by infectious agents, drug effects, metabolic conditions or mechanical complications of surgery. In this study, the presence of gastrointestinal signs was statistically correlated with KIPyV infection. Since gastrointestinal complications are often associated with worse outcomes, determining the cause may suggest whether to initiate an antiviral therapy and the duration of treatment. Reactivation of HPyVs might influence treatment efficacy and have a synergistic effect on the reactivation of other viruses. Thus, studying in depth the contemporary presence of HPyVs might be significant and improve the understanding of the pathogenic potential of these viral species in immunocompromised hosts.

NCCR structure organization is important because of the association of the rearranged variants with specific human diseases, such as PML for JCPyV and nephropathy for BKPyV [52–55]. In light of that, we investigated the sequence variability of the HPyVs NCCRs in the gastrointestinal tract of hematological patients, in effort of advancing our understanding of HPyVs biology. The NCCRs isolated from JCPyV-positive samples showed an archetype structure with nucleotide changes corresponding to known sequence polymorphisms [56–58]. A G217 to A nucleotide transition was identified in the NCCR sequences of patients with the European genotype, corroborating that this mutation represents a common feature of the European strains [58]. Similarly to JCPyV, alignment of BKPyV NCCRs showed an archetype structure with point mutations that did not involve any cellular transcriptional factor-binding sites. Alignment of KIPyV, WUPyV and MCPyV NCCRs showed a high degree of sequence stability, suggesting that rearrangements in this anatomical site are probably rare and providing also an explanation for the low viral load measured. In fact, it is well known that NCCR rearrangements can increase viral replication and influence gene expressions and virulence properties [59].

HPyVs VP1 phylogenetic analysis showed that all isolates grouped into a main cluster together with corresponding reference strain. Since the circulation and the genetic evolution of HPyVs were influenced by virus infectivity and/or virus antigenic variability, monitoring amino acid changes could be useful to improve the understanding of the epidemiological and clinical features of HPyVs.

In conclusion, our results add new information on the prevalence, replication and persistence of HPyVs in the gastrointestinal tract, although the duration of HPyVs shedding in fecal matter after primary infection has not been settled yet. Larger studies that will include serial sample collections, to control for intermittent excretion, will be necessary to clarify this point. Moreover, cell culture systems will be useful to study the effects of NCCR variants on viral replication and virulence in this anatomical site [60].
